# Instrument Pointer Recognition Scheme Based on Improved CSL Algorithm

**DOI:** 10.3390/s22207800

**Published:** 2022-10-14

**Authors:** Hailong Liu, Jielin Wang, Bo Ma

**Affiliations:** 1 School of Electronic Engineering, Xi’an Shiyou University, Xi’an 710065, China; 2 Key Laboratory of Shanxi Province for Gas and Oil Well Logging Technology, Xi’an 710065, China

**Keywords:** YOLOv5, object detection, CSL, binary coding, angle recognition

## Abstract

The traditional pointer instrument recognition scheme is implemented in three steps, which is cumbersome and inefficient. So it is difficult to apply to the industrial production of real-time monitoring. Based on the improvement of the CSL coding method and the setting of the pre-cache mechanism, an intelligent reading recognition technology of the YOLOv5 pointer instrument is proposed in this paper, which realizes the rapid positioning and reading recognition of the pointer instrument. The problem of angle interaction in rotating target detection is eliminated, the complexity of image preprocessing is avoided, and the problems of poor adaptability of Hough detection are solved in this strategy. The experimental results show that compared with the traditional algorithm, the algorithm in this paper can effectively identify the angle of the pointer instrument, has high detection efficiency and strong adaptability, and has broad application prospects.

## 1. Introduction

In most commercial and civil scenarios, pointer meters have been replaced by simpler and more convenient digital meters, but in industrial scenarios with harsh environments, digital meters are often difficult to work normally and cannot effectively monitor the results. Therefore, pointer meters are still widely used in industrial scenarios due to their advantages of stability, strong anti-interference ability, and easy cable installation. However, the problem of reading storage of pointer meters has not been effectively solved, resulting in the existing pointer meters being unable to meet the urgent needs of intelligent development of industrial production. The problem is particularly prominent in fieldwork environments such as sports vehicle systems or oil drilling engineering.

With the development of deep learning, the research on automatic identification of instruments based on deep learning has gradually been carried out. Wan Jilin et al. introduced the Faster R-CNN method to detect the meter and pointer area, and the Dice function of U-Net is constructed to solve the problem of classification imbalance and improve the accuracy and practicability of small targets in complex images [1]. Aiming at the problem of insufficient generalization of the current detection methods, Ma Bo et al. used adaptively extracted key features as prior knowledge to generate virtual samples to optimize the recognition effect and increase the robustness in complex situations but did not realize the problem of dial correction [2]. Chen Mengchi et al. used QR codes to locate and correct the perspective transformation, so they were able to overcome the problem of image distortion caused by the inclination of the shooting angle [3]. Zhou Dengke et al. used generalized least squares to perform ellipse fitting on the key points extracted by the convolutional neural network and realized tilt and rotation correction by using perspective transformation and calculation of the key symmetry point of the central axis of the instrument [4]. Aiming at the problem of the large number of parameter calculations in deep learning, Li Huihui et al. used the improved MobileNetV2 network mixing combined with Hough detection, compared with ResNet, the result reduces 90.51 percent of the parameters and 92.40 percent of the calculation, which is helpful for further deployment in mobile or embedded devices [5]. Summarized the traditional scheme, Shen Weidong et al. used the SSD network to locate the position of the instrument in the complex background, and used the multi-scale Retinex algorithm to enhance the HSL color space image. Finally, Canny edge detection 42 and Hough transformation are used to obtain the pointer tilt angle [6]. Xu Li et al. proposed an iterative maximum inter-class variance algorithm to optimize the pointer extraction under different illuminations and added constraints on the Hough transform to achieve a recognition rate of 95 [7].

However, the traditional strategies of classic deep learning algorithms in pointer meter recognition are not suitable for the embedding of real-time monitoring systems. Although the detection model has been optimized at present [8,9], the image processing such as illumination, rotation correction, and Hough detection algorithm still requires a lot of time and is inefficient. Therefore, the traditional deep learning scheme is still limited in practical applications.

A new strategy of introducing the CSL algorithm into the YOLOv5 framework to detect the rotating target of the meter and the pointer is presented in this paper, which can simplify the detection steps and realize the direct recognition of the angle of the pointer and pointer meter. Binary encoding is used to improve the original encoding method of CSL, and the pre-cache mechanism is introduced into the algorithm to reduce the number of parameters and the calculation of the original algorithm in this strategy, then the accuracy of angle detection is improved and the angle interaction problem existing in the original algorithm is solved. The experimental results show that the scheme is not only less sensitive to light interference, but also can effectively solve the problem of rotation correction. The scheme has strong adaptability to the detection of different instruments, and the detection speed is far faster than the traditional scheme.

## 2. Instrument Image Features and Recognition Model

### 2.1. Traditional Identification Technology and Its Problems

**Figure 1:**
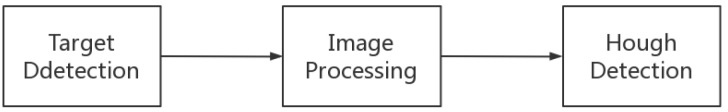
Traditional instrument identification process.

The traditional instrument identification method is generally divided into three steps [10,11,12,13]: firstly, target detection to remove the background; secondly, image preprocessing to adjust the light shadows and correct the meter rotation; finally, Hough detection to obtain the angle and calculate the reading. The scheme is shown in Figure 1.

Pointer parameter images typically suffer from three flaws in unstable environments such as movement scenes or in the wild:

(1) The instrument image background is complex. Instruments are often used in outdoor environments with complex and diverse backgrounds, such as pipelines on oil sites, drilling rigs, and other complex scenes.
(2) The instrument image has uneven light and shadow. Whether it is an indoor or outdoor scene, the light received by the instrument image is too dark, too bright, or uneven in brightness, which will increase the difficulty of identification.
(3) Instrument image rotation. Due to equipment reasons, it cannot be guaranteed that the instrument is installed in the standard position, and the picture of the instrument in the camera may be rotated or even reversed.

In order to solve the problems above, the traditional pointer instrument image recognition technology usually needs to spend a lot of time for additional image preprocessing, this method is inefficient and slow, resulting in a serious waste of resources and it cannot meet the real-time requirements of industrial production [14].

### 2.2. Intelligent Identification Technology of Pointer Meter Based on Improved Circular Smooth Label Algorithm

**Figure 2:**
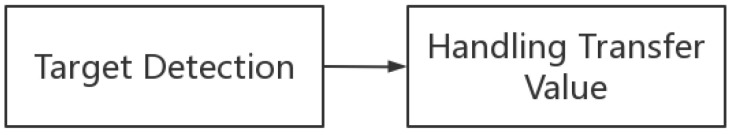
Identify readings based on CSL detection.

Considering the characteristics of common instrument images, an automatic recognition scheme for YOLOv5 instrument pointers based on the CSL (circular smooth label) algorithm is designed. The process is shown in Figure 2.

As shown in Figure 2, a target detection algorithm to detect the angle of the pointer and the meter directly is proposed in this paper. The angle is used to calculate the relative angle difference, and the information needed for the meter identification is obtained directly from the value of the relative angle in one step in this new algorithm. This paper will use the target detection algorithm to directly detect the angle of the pointer and the meter, use the angle to calculate the relative angle difference, and directly pass the value to obtain the information needed for meter identification in one step.

## 3. Introduction and Defects of Circular Smooth Label Algorithm

### 3.1. Circular Smooth Label Algorithm

The CSL (circular smooth label) algorithm is an angle prediction method without boundary problems. The current problems of angle prediction based on 97 regression methods can be summarized as: the ideal prediction result exceeds the initially defined range and produces a large loss. Dr. Yang proposed the CSL algorithm and limited the range of the prediction results to reduce the angle classification error so that the IOU loss of the prediction box and anchor box is minimized to improve the target result [15].

### 3.2. Use of Circular Smooth Label Algorithm

#### 3.2.1. Circular Smooth Label Algorithm Labeling Method

A Gaussian function is used as a window function for angle classification by comparison in CSL, the original angle is calculated separately and brought in the corresponding label for angle classification. A similar idea of sliding labels is also used in borderless object detection [16]. The calculation method is shown in Formula (1):

(1) $$\mathrm{CSL}=f\left(x\right)=\left\{\begin{array}{c} e^{\frac{-{\left(x-\theta \right)}^{2}}{2r^{2}}},\theta -r\leq x\leq \theta -r\\ 0,\;\mathrm{otherwise} \end{array}\right.$$

#### 3.2.2. Feasibility Analysis of Circular Smooth Label Algorithm

The CSL algorithm converts angle recognition from a regression problem to a classification problem. From continuous to discrete, there will be a loss of accuracy. The algorithm divides the angle into integer categories, which makes it impossible to predict non-integer angles. In the case of integer classification, the classification interval can be assumed as ω, and the angle maximum accuracy loss and average loss are shown in Formulas (2) and (3):

(2) $$\begin{array}{cc} \; & \mathrm{Max}(\mathrm{loss})=\omega /2 \end{array}$$

(3) $$\begin{array}{cc} \; & E\left(\mathrm{loss}\right)=\int_{0}^{\omega /2}x*\frac{1}{\omega /2-0}\mathrm{dx}=\omega /4 \end{array}$$

**Figure 3:**
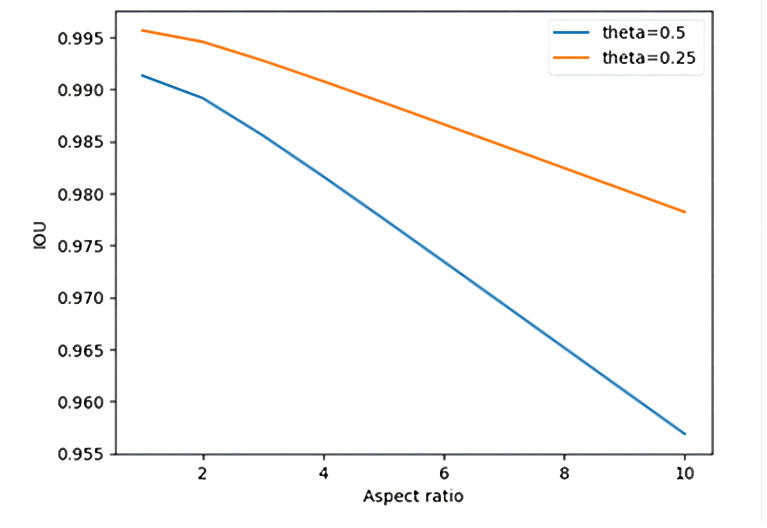
IOU loss under different aspect ratios.

We take ω as the smallest integer, 1 as an example, and the center point of the prediction frame as the rotation. The maximum loss deviation and average loss deviation of the IOU under different aspect ratios of the image are shown in Figure 3.

As shown in Figure 3, as the aspect ratio of the detected target frame increases, the IOU loss will increase. If ω takes the smallest integer 1 and the aspect ratio is 10, the average loss deviation of IOU generated by the picture is 0.022, and the maximum loss deviation is only 0.043. The IOU loss of angle participation is within the allowable range, so it is feasible to perform angle prediction in a classification manner.

#### 3.2.3. Limitations of Circular Smooth Label Algorithm

The original intention of the CSL algorithm is to reduce the IOU loss of the anchor frame by classification to further improve the accuracy of target detection. It does not pay much attention to the prediction ability of the angle. Based on many experiments, the CSL algorithm has two limitations.

(1) Classification limitations

CSL is mainly classified by integer angles, so it cannot be more precise. When the classification interval is further reduced to improve the prediction accuracy, the prediction classification parameters and the calculation amount will increase, which has certain limitations.

(2) Ambiguous orientation

The CSL algorithm does not take the interactivity of angles into account. The algorithm uses the −90 to 90-degree range of the long-side notation angle notation to represent −180 to 180 degrees, but the pointer angle generally requires an exact and fixed value.

## 4. Circular Smooth Label Algorithm Improvement

### 4.1. Improvement of Classification Limitations

The classification interval determines the prediction accuracy. As the classification interval decreases, the classification category increases, and the accuracy increases, too. Theoretically, the smaller the classification interval, the better the measurement result. However, in fact, too many classification categories will lead to an increase in the prediction parameters and increase the complexity of classification calculation.

Binary coding [17] is used to improve the coding of CSL classification and the classification category is represented by binary coding. Taking four categories as an example, the encoded annotation types are shown in Table 1.

Under binary coding, multi-objective classification can be regarded as a simple binary coding classification problem, but the number of classification types will be limited to $2^{n}$. For example, the classification category has 512 classes. The angular interval of the classification is 0.352, the IOU loss deviation is almost negligible and a certain accuracy is added.

Under this classification, the CSL algorithm needs to use 512 parameters to represent angles, but the binary-coded CSL algorithm only needs eight parameters to represent 512 types of angles. During prediction, the angle parameter *T_n_*, the total angle participation parameter *P_n_* and the parameter calculation amount *C_n_* are calculated by the following formulas:

(4) $$\begin{array}{c} T_{n}=Anchor_{-}num\times Prarmeter \end{array}$$

(5) $$\begin{array}{c} P_{n}=\;{\mathrm{Channel}}_{\mathrm{out}}\times \;\ker\;\mathrm{nelsize}\;{}^{2}+T_{n} \end{array}$$

(6) $$\begin{array}{c} C_{n}=2\times \;{\mathrm{Channel}}_{\mathrm{out}}\times l\times \;\ker\;\mathrm{nelsize}\;{}^{2}\times \;\mathrm{pixel}\;\mathrm{num}\; \end{array}$$

(7) $$pixel_{-}num=Channel_{in}\times sum\left(h_{i}\times w_{i}\right)$$

Using YOLOv5 as the piggyback model for this algorithm, The number of prediction boxes (*Anchor_num*) is 9. “*Parameter*” represents the number of parameters involved in the angle calculation, The number of channels output by the prediction layer is 256, and the convolution kernel size is 3. The prediction layer feature image size includes (76, 76, 256), (38, 38, 256), and (19, 19, 256). The parameters and calculation amount of the CSL algorithm under YOLOv5 and the improved CSL algorithm are shown in Table 2.

As shown in Table 2, compared with the target detection in the original regression method, the improved algorithm uses fewer relative to the CSL algorithm in terms of the number of parameters and the amount of computation, which actually reduces the time by about 70 percent in the actual training and testing.

### 4.2. Improvement of Directional Ambiguity

When making angle predictions, there are often cases where the actual angle differs from the predicted angle by about 180 degrees. This is due to the interaction of the angles. As shown in the figure, the real angle should be angle “b”, but in the CSL algorithm, the angle is limited to −90 degrees to 90 degrees, and the predicted result is angle “a”, which is 180 degrees different.

**Figure 4:**
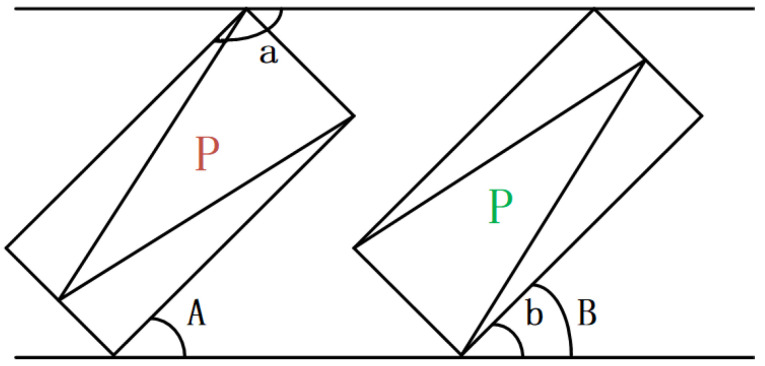
Angle interactivity.

In Figure 4, the pointer green “P” points to the upper half area, that is, when the pointer angle is in the range [0, 180), the predicted angle “B” and the actual angle “b” are the same; however, when the pointer red P points to the lower half area, that is, when the pointer angle is in the range [0, −180), the actual angle is “a” but the predicted angle is “A”, and the difference between the two angles is 180 degrees. Therefore, the angle transformation should be introduced.

In Figure 4, if the pointer angle is in the range [0, 180), the predicted angle B and the actual angle b are the same angle when the pointer green P points to the upper half area. When the pointer red P points to the lower half area, if the pointer angle is in the range [0, −180), the actual angle is a but the predicted angle is A and the difference between the two angles is 180 degrees. In this case, angle transformation is required.

As shown in Table 3 relative angle predictions, the predicted value is shown by “Pred(a,b)”, “a” represents the predicted dial angle result, “b” represents the predicted pointer angle result, “Error” represents the relative error with the actual angle, and “T/ F” represents the prediction result of the relative angle.

Due to the interactivity of the angle, setting the angle label range between −180 and 180 is not ideal for angle prediction due to the interactivity of angles. Therefore, the idea of frame angle cache is proposed, and the angle of the previous frame image is cached. Thresholds are set to ensure the continuity of angle changes. If the angle jump exceeds the threshold, it can be considered that the predicted angle and the real angle have interactively transformed, and then adjust the forecast angle.

## 5. Implementation of the Algorithm

### 5.1. YOLOv5 Rotating Target Detection Based on Circular Smooth Label Algorithm

At present, there are three approaches for object detection with bounding boxes: one-step SSD [18], YOLO, and two-step Faster R CNN [19] frameworks. However, due to the real-time nature of instrument identification, a one-step network framework needs to be selected for target detection, and the target detection includes small pointer targets, which requires a relatively high-precision method for target detection. Therefore, the YOLOv5 algorithm is selected as the instrument identification detection framework.

#### 5.1.1. Introduction to the Advantages of YOLOv5

(1) Using the Mosaic method [20] to enhance the data, through random cropping, scaling, and random stitching of four pictures, the detection effect of small targets is strengthened.
(2) Using the adaptive anchor box, each time the optimal anchor box value of different training sets is calculated, the training can converge faster.
(3) Focus structure, slice the input feature image, improving the model detection speed from the amount of calculation and the number of parameters.
(4) Using GIoU loss as the bounding box upfront loss function, CIoU loss as the bounding box late loss function, and using DIoU loss in the process of NMS [21] not only improves the convergence speed and performance of the model but also enhances the ability to detect occluded overlapping objects.

#### 5.1.2. The Improved Circular Smooth Label Algorithm

The CSL algorithm after using binary encoding is still the classification algorithm for angles. During the training process of YOLOv5, we should encode and decode the angle information of the annotation information. The pseudocode for the encoding and decoding process during training is as follows in Algorithms 1 and 2.
**Algorithm 1** Binary encode**Input:** label before encode, Angle interval ω**Output:** label encoded    **for** label.θ in label **do**       θ=label.θ;       delete label.θ;       theta_encode =Bin(−Round(θ−90)/ω);       list_theta = list(theta_encode);       **for** *i* in List_theta **do**           label.append (i);       **end for**    **end for**    **return** label;

**Algorithm 2** Binary decode
**Input**: model predict result, Angle interval ω**Output:** result decoded    **for** line in result **do**        pred $=\mathrm{line}[\log_{2}\left(180/\omega \right):-1]$;        θ=90−ωInt(Round(Sigmoid(pred)));        Delete pred from line;        Line.Append(θ);    **end for**    **return** result;


The pseudocodes in Algorithms 2 and 3 represent the encoding and decoding operations used for training, respectively. The input unencoded angle information is the normal angle information marked by the field side notation, and the angle interval is the passing angle.

For the predicted angle, only predicted values for 0–180 degrees exist and the predicted value corresponds from −180 to 180 and the threshold is 90. The pseudocode of the algorithm is as follows in Algorithm 3.

In Algorithm 4, since the predicted angle can only recognize the angle of 0–180 degrees, the boundary threshold of 90 degrees should be used as the threshold for the 226 algorithm’s angle exchange directly. When the absolute value of the predicted angle value minus the angle value of the previous frame buffer is greater than 90 degrees, the predicted angle can be considered to have an angle misprediction. In this case, the angle needs to be exchanged, and the obtained dial and pointer angles are cached to replace the cached frame angle for the next prediction comparison.
**Algorithm 3** Angle transform   Using the initial state value to initialize the cached angle list.**Input:** cached angle list, predict angle list**Output:** cached angle list, relative angle   para_angle c0= cached angle list[0]   po_angle c1= cached angle list[1]   para_angle p0= predict angle list[0]   po_angle p1= predict angle list[1]   **if** −90⩽c0−p0⩽90 **then**      cached angle list[0] =p0;   **else**      p0=p0−180;      cached angle list[0] =p0;   **end if**   **if** −90⩽c1−p1⩽90 **then**      cached angle list[1] =p1;   **else**      P1=p1−180;      cached angle list[1] =p1;   **end if**   relative angle =p1−p0   **Return** cached angle list, relative angle

**Algorithm 4** Data augmented
**Input:** The original images**Output:** Augmented images & Augmented labels    Label ≪ using labelimg get json labels;    Read The original images;    **for** *i* **in** 180 **step** 3 **do**        Images ≪ Rotate the image i degrees;        Labels ≪ Rotate the label i degrees;        Write images and labels in Augmented images files & Augmented labels files;    **end for**    **for** label in Augmented labels files **do**        **if** ∃num∈(x1,y1,x2,y2,x3,y3,x4,y4) in label out of boarders **then**            delete label and pair image        **end if**    **end for**    **for** label in Augmented labels files **do**        label ≪ normalize label;        Yolo label ≪ minAreaRect(label)        **if** w<h **then**            w,h=h,w;            θ=θ+90;        **end if**    **end for**


### 5.2. Fit Calibration Method

In actual detection, the dial is often tilted or laid flat. The traditional technology needs to correct the tilt of the dial before proceeding to the next step of pointer recognition.

Using the scheme of this paper to detect the instrument recognition target will obtain two angles. One is the rotation angle of the current meter—offset. The other is the angle of the pointer relative to the horizontal and subtract the meter angle from the last pointer angle obtained. The tilt correction process to obtain the angle of the pointer relative to the dial can be avoided, thus the detection speed can be improved greatly. It can directly avoid the tilt correction process to obtain the angle of the pointer relative to the dial, which greatly speeds up the detection speed.

## 6. Experimental Results

### 6.1. Data Annotation and Data Augmentation

About 1000 instrument image data captured on site and searched on the Internet were selected for data annotation augmentation. The pseudocode of the data annotation augmentation process is as follows in Algorithm 4.

Firstly, the json file data annotation is used to get the initial data and rotate the image at multiple angles. Secondly, the annotation information recorded in the json file is utilized to rotate the annotation frame for data augmentation, and the information beyond the boundary are filtered. Thirdly, the center point and the angle information of the opencv representation are obtained. Finally, the annotation information is converted into long-side notation and the data is normalized. As a result, about 18,362 YOLO annotations with angular long-side notation are obtained.

### 6.2. Algorithm Verification

#### 6.2.1. Object Detection Accuracy

In the algorithm verification process, considering the problem of frame labeling caused by the tilt of the meter and the pointer, in this paper we choose to separate the detection accuracy of the meter and the pointer to discuss the classification of the three algorithms. Table 4 separate discussion of the detection accuracy of meter and pointer under various algorithms.

The table represents the average precision of the gauges and pointers on the training and validation sets, respectively. It can be seen from the table that, as for the detection accuracy of the instrument, the original Yolov5 has the best mesh results, and the CSL and CSL_B detection accuracy is not better than it. As for pointers, the detection accuracy of the YOLOv5 model with CSL or CSL_B is higher than the original model. It proves that target detection with added rotation detection is better for target detection with a relatively large aspect ratio. Additionally, objects with oblique angles with larger aspect ratios have a smaller proportion of features in the original calibration frame. Therefore, rotating object detection reduces feature loss and makes prediction more accurate.

#### 6.2.2. Angle Error Calculation

**Figure 5:**
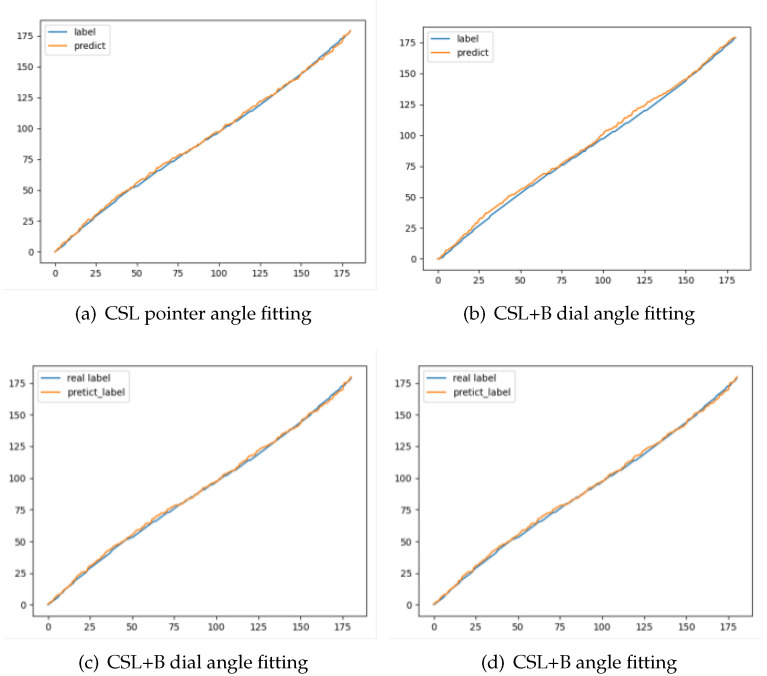
Annotation and prediction angle fitting curve.

In the experiment, there are two types of angle errors. One of the artificial angle errors is in the process of calibrating the label, the other is the machine angle error during prediction. It requires the calculation of machine angle error for verification, but human error cannot be avoided. Therefore, in this paper, we take 1 degree as the artificial error threshold and consider that the angle prediction is correct if the error between the predicted value and the calibration value is between (−1, 1). If the threshold is exceeded, the angle error value needs to be calculated. The pointer and meter fitting curves of the CSL algorithm and the improved algorithm are shown in Figure 5.

Figure 5a,b show the pointer, meter angle labeling, and prediction fitting curve under the CSL algorithm, respectively. Figure 5c,d are meter labeling and prediction fitting curves. In the figure, it can be seen from the figure that the detection result of traditional rotating target detection for discs with a small aspect ratio is much worse than that of a pointer with a large aspect ratio. The improved algorithm is not much different from the prediction results of the CSL algorithm in the prediction of integer angles, but the prediction error for non-integer results is smaller.

**Figure 6:**
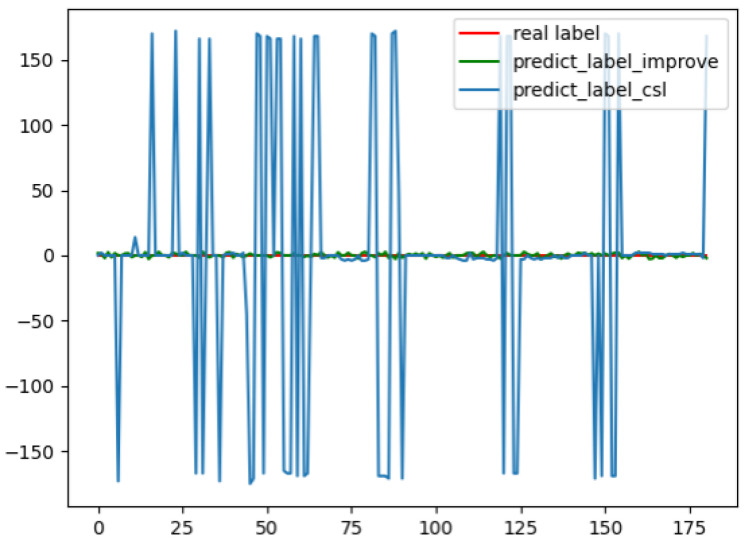
Annotation and prediction angle fitting curve.

The error curve between the true relative angle value and the prediction result after Algorithm 4 is shown in Figure 6.

In Figure 6, the blue curve is the angle error of the original CSL algorithm, the green curve is the error of the improved CSL algorithm, and the red curve is the error 0 baseline. In the picture, we can obtain the CSL algorithm that has not been processed by Algorithm 4 has a maximum error of ±180 degrees relative to the baseline. The interaction of angles is not considered so that the predicted angles are between [0, 180]. At this time, the detection results of the pointer and the meter do not match, and the relative angle deviation is about 180. However, after Algorithm 4, the prediction result will be corrected according to the result of the previous frame, which is very stable.

In addition, this paper carries the improved CSL algorithm on several different detection models for comparison. The detection rate and accuracy are shown in Table 5.

YOLOv5, due to the advantages of its own framework improvement, the original detection effect is relatively good. This paper compares the detection accuracy of the common models equipped with the CSL algorithm. After the comparison of various models equipped with the CSL rotation detection algorithm, the YOLOv5 detection model has higher accuracy in pointer and meter recognition. Additionally, it is more dominant in industrial applications.

### 6.3. Algorithm Verification

#### 6.3.1. Algorithm Selection Design

Since the traditional algorithm needs to process the image and then perform Hough detection to obtain the pointer angle, it is not suitable for the basic detection network to select the same YOLOv5 for comparison. SSD is selected as the target detection network of the traditional algorithm and made backbone replaced with the MobileNet [25] network to speed up detection. Then we use the adaptive Retinex [26,27] to adjust the light and rotation correction using least squares and perspective transformation [28] from the literature [4]. As for Unet [29] segmentation, the detection pointer needs to classify each pixel of an image. As for its consuming trait, the faster Hough detection [30] is selected to obtain the position of the center of the circle and the angle information of the pointer, and finally, the pointer reading is obtained.

#### 6.3.2. Comparison of Detection Effects

Since the SSD network has a very high missed detection rate for small targets, the pointers to small targets are basically undetectable. However, YOLOv5 is still very friendly to small targets [31]. Therefore, this section does not compare the detection accuracy, the problem of image jitter is generally solved on the hardware camera side, and the jitter problem is not considered in this section.

The system environment of the test results includes Windows 10 Education Edition, Inter (R) Core (TM) i9-10900X CPU @ 3.70 GHz processor, memory (RAM): 64.0 GB, system type: 64-bit operating system, and GPU: 2080Ti.

**Figure 7:**
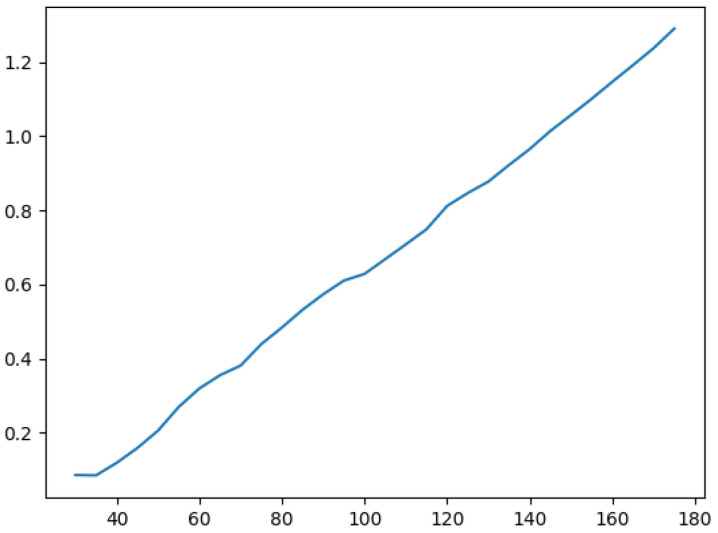
Influence of Gaussian surround scale on time.

Since the image processing time of the Retinex feature enhancement algorithm is related to the image quality and the Gaussian surround scale, the real adaptive Retinex algorithm test consumption time is not stable with different lighting, as shown in Figure 7. Therefore, the more stable single-scale Retinex algorithm is selected for comparison, and the Gaussian surround scale is fixed as 80, which is commonly used, and the average processing time obtained is used as the comparison time of the illumination correction algorithm.

In Table 6, rotation correction and lighting correction are time-consuming in practice, and the average optimal detection frame rate in an ideal environment is 1.4 fps. It is inconvenient whether it is in real-time monitoring or storage processing. The improved CSL_B algorithm directly obtains the angle from the target detection model, and the sensitivity of illumination is very low. The angle is processed during detection, and the detection is 26 fps, which can fully meet the real-time requirements.

In the comparison of traditional algorithms, it is found that the traditional Hough detection has the disadvantage of weak adaptive ability, as shown in Figure 7. For Hough detection of different instruments, it may be necessary to set corresponding thresholds to ensure the detection of circles, circle centers, and straight lines. Otherwise, the detected results will have great deviations. In this paper, the algorithm target detection obtains results in one step and has stronger adaptive ability and stability.

#### 6.3.3. Test Result 1

To test the feasibility of the proposed scheme, the error detection of the test results is carried out under normal conditions, uneven illumination, and a certain rotation angle. The scale range of the detected instrument is 0.0 to 0.6 MPa, and the pressure gauge is evenly scaled. The test results are shown in Table 7, Table 8 and Table 9.

As shown in the tables, in this paper, the measurement accuracy is set to two decimal places to magnify the similarities and differences between the measurement results. The detected values obtained under ideal conditions in Table 7 are basically consistent with the real values, with occasional errors, and the error value does not exceed 3.4 percent. After adding an appropriate amount of uneven illumination, the results remain unchanged, which also shows that the algorithm is not sensitive to the influence of illumination.

In Table 9, unquantified rotation due to machine detection and manual rotation errors will slightly increase the detection error. However, from the data point of view, the error value can be controlled within the range of 5 percent, and it still shows a very stable prediction result, which basically meets the use of drilling sites.

#### 6.3.4. Test Results 2

**Figure 8:**
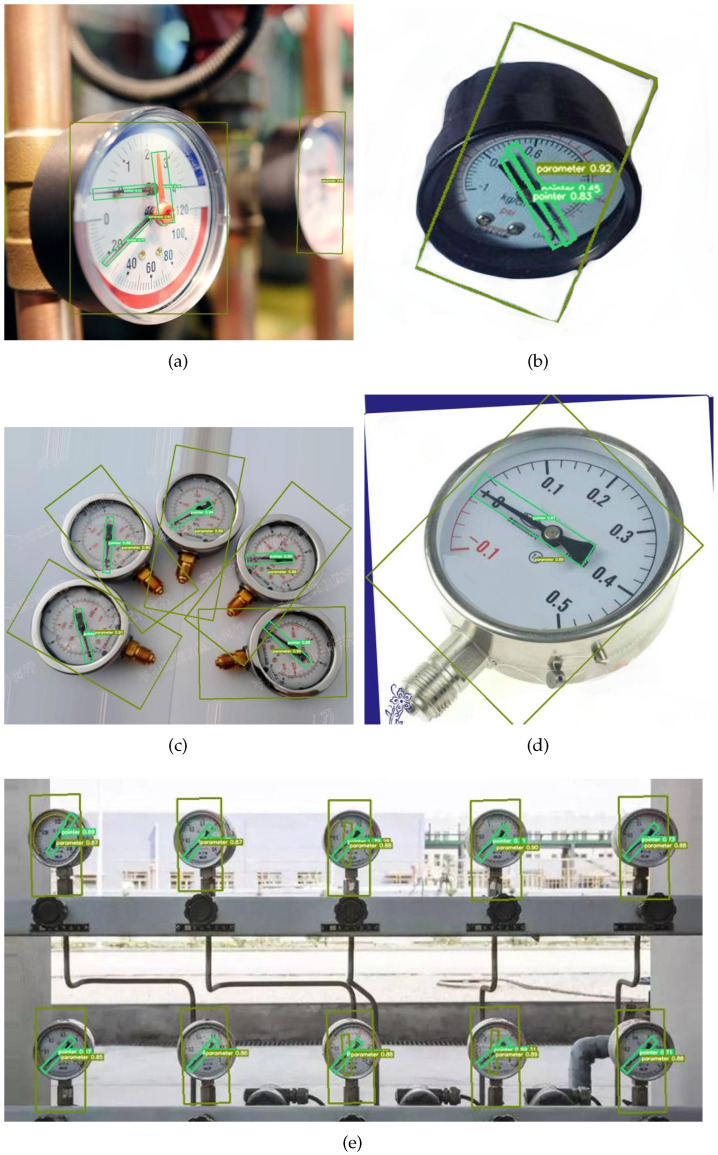
Annotation and prediction angle fitting curve.

An example of the detection results of the algorithm part is shown in Figure 8 and the detection result is better.

Figure 8a,b show the results of the algorithm detecting the instrument images captured in different directions. Additionally, Figure 8a includes multi-pointer detection. This case shows the algorithm can effectively solve the problem of multi-pointer detection and can effectively detect and identify the distortion meters photographed from different angles.

Figure 8c shows the detection and recognition results of the algorithm in multi-target detection and instruments under different rotation angles. It can be seen that the algorithm effectively framed each meter and pointer without any missed detection or false detection.

Figure 8d shows the detection results of the instrument image with a certain rotation angle and a certain distortion. The results show that the algorithm can effectively solve the problems of distortion and rotation interference.

Figure 8e shows the algorithm detection results of multiple targets and small targets. It can be seen from the results that there is no missing detection in the algorithm detection, indicating that the algorithm still performs well in small target recognition.

From the example graph of the detection result of Figure 8, we can see that the algorithm can effectively detect pointers and the algorithms enable efficient detection of pointers and gauges. Meanwhile, the algorithm can also effectively detect distorted, rotated meter images and even small target meter images.

The detection algorithm proposed in this paper has a strong anti-interference ability and can meet the detection of small targets, and has strong practicability in instrument detection and identification.

## 7. Conclusions

A new strategy for an instrument pointer recognition scheme is proposed in this paper, two defects of the CSL algorithm in angle detection are indicated and the 368 algorithm has been improved by using the strategies of binary encoding and threshold contrast preset and cached. The problem of the CSL algorithm is solved, and the results of the algorithm are also verified. The improved CSL algorithm is introduced into the OBB target detection of the YOLOv5 model, then the direct detection of the pointer angle is realized, and the one-step intelligent identification of the pointer-type meter readings is completed. Compared with traditional schemes, the tedious process of image preprocessing is avoided, the effects of light and shadow are overcome, and the rotational correction process to the instrument image is eliminated. Additionally, the problem of the insufficient adaptation ability of Hough detection is also addressed, and the ability of the improved algorithm scheme to detect small target instruments is also greatly improved, which can meet the requirements of industrial production in accuracy and speed. This new strategy has an important application value to the intelligent development of industrial production. 

## Figures and Tables

**Table 1:** Binary coded annotation type.

Theta	−90	−45	0	45
Class num	0	1	2	3
Onehot label	0001	0010	0100	1000
Binary code	00	01	10	11

**Table 2:** Comparison of parameter calculations.

Method	Tn	Pn	▴Pn	Cn	▴Cn
Regress	9	46.6 M	-	252.7	-
CSL	4608	57.2 M	22.7 percent	441.3	74.6 percent
Bin+CSL	72	46.8 M	0.4 percent	261.1	3.4 percent

**Table 3:** Relative angle forecast.

Pred	(0, 0)	(0, 1)	(1, 0)	(1, 1)
Error	0	180	−180	0
T/F	T	F	F	T

**Table 4:** Relative angle forecast.

Method	$Pa\_t_{AP}$	$Po\_t_{AP}$	$Pa\_v_{AP}$	$Pa\_v_{AP}$
Yolov5	99.2 percent	95.7 percent	97.3 percent	87.6 percent
Yolov5+CSL	98.6 percent	96.3 percent	94.2 percent	90.3 percent
Yolov5+CSL_B	97.3 percent	94.8 percent	92.6 percent	88.5 percent

**Table 5:** Comparison of detection results of different models.

Method	SSD	RCNN [22]	RRPN	Faster RCNN
MAP	63.21 percent	72.01 percent	76.04 percent	88.32 percent
Method	RRD [23]	RoI-Transformer [24]	RetinaNet-R [14]	Yolov5
MAP	85.64 percent	89.12 percent	90.23 percent	93.3 percent

**Table 6:** Relative angle forecast.

Method Detection	Target Correction	Lighting Correction	Rotation Detect	Hough Duration	Total Rate	Frame
Traditional	36 ms	468 ms	230 ms	4.9 ms	739 ms	1.4 fps
CSL_B	38 ms	0 ms	≈0 ms	0 ms	38 ms	26 fps

**Table 7:** Test result table.

Manual measurement of the true value	0.00	0.10	0.15	0.20	0.25	0.30
The value calculated by the algorithm in this paper	0.00	0.10	0.15	0.20	0.24	0.30
Manual measurement of the true value	0.35	0.40	0.45	0.50	0.60	/
The value calculated by the algorithm in this paper	0.37	0.40	0.45	0.51	0.60	/

**Table 8:** Measurement results under uneven lighting.

Manual measurement of the true value	0.00	0.10	0.15	0.20	0.25	0.30
The value calculated by the algorithm in this paper	0.00	0.10	0.15	0.20	0.24	0.30
Manual measurement of the true value	0.35	0.40	0.45	0.50	0.60	/
The value calculated by the algorithm in this paper	0.37	0.40	0.45	0.51	0.60	/

**Table 9:** Random angle rotation measurement results.

Manual measurement of the true value	0.00	0.10	0.15	0.20	0.25	0.30
45 degree test results	0.02	0.11	0.15	0.20	0.26	0.31
90 degree test results	0.00	0.11	0.15	0.20	0.24	0.31
−60 degree test results	0.00	0.10	0.16	0.20	0.24	0.33
Manual measurement of the true value	0.35	0.40	0.45	0.50	0.60	/
45 degree test results	0.35	0.43	0.44	0.51	0.60	/
90 degree test results	0.35	0.41	0.46	0.50	0.60	/
−60 degree test results	0.34	0.41	0.44	0.50	0.58	/

## Data Availability

Not applicable.
